# Pulmonary blood volume indexed to lung volume is reduced in newly diagnosed systemic sclerosis compared to normals – a prospective clinical cardiovascular magnetic resonance study addressing pulmonary vascular changes

**DOI:** 10.1186/1532-429X-15-86

**Published:** 2013-09-25

**Authors:** Mikael Kanski, Håkan Arheden, Dirk M Wuttge, Gracijela Bozovic, Roger Hesselstrand, Martin Ugander

**Affiliations:** 1 Department of Clinical Physiology, Lund University and Lund University Hospital, Lund, SE-221 85 Lund, Sweden; 2 Department of Rheumatology, Lund University and Lund University Hospital, Lund, SE-221 85 Lund, Sweden; 3 Department of Radiology, Lund University and Lund University Hospital, Lund, SE-221 85 Lund, Sweden

## Abstract

**Background:**

Pulmonary involvement, manifested as pulmonary arterial hypertension or pulmonary fibrosis, is the most common cause of death in systemic sclerosis (SSc). We aimed to explore the feasibility of detecting early pulmonary involvement in SSc using recently developed non-invasive quantitative measures of pulmonary physiology using cardiovascular magnetic resonance (CMR).

**Methods:**

Twenty-seven SSc patients (9 men, 57 ± 13 years) and 10 healthy controls (3 men, 54 ± 9 years) underwent CMR to determine the pulmonary blood volume (PBV) and the PBV variation (PBVV) throughout the cardiac cycle. Patients underwent Doppler echocardiography, high-resolution computed tomography (HRCT), and pulmonary function testing by spirometry. Comparisons were performed using the unpaired t-test and linear regression analysis was performed with Pearson’s correlation coefficient (r).

**Results:**

Compared to healthy controls, the PBV indexed to lung volume (PBVI) was lower in patients (16 ± 4 vs 20 ± 5%, p < 0.05). There was no difference in PBV (466 ± 87 vs 471 ± 122 mL, p = 0.91) or PBVV/stroke volume (45 ± 10 vs 40 ± 6%, p = 0.09). There were no significant correlations between PBVI and pulmonary artery pressure estimated by Doppler (p = 0.08) the lung’s diffusion capacity for carbon monoxide (DL_CO_) (p = 0.09), vital capacity (p = 0.45), or pulmonary fibrosis by HRCT (p = 0.74).

**Conclusions:**

This study is the first to measure the PBV in humans using CMR. Compared to healthy controls, newly diagnosed SSc patients have a reduced amount of blood in the pulmonary vasculature (PBVI) but unchanged pulmonary vascular distensibility (PBVV/stroke volume). PBVI is unrelated to DL_CO_, pulmonary artery pressure, vital capacity, and the presence of pulmonary fibrosis. PBVI may be a novel parameter reflecting vascular lung involvement in early-stage SSc, and these findings may be consistent with pathophysiological changes of the pulmonary vasculature.

## Background

The diagnosis of systemic sclerosis (SSc) entails a 10–15% lifetime risk of developing pulmonary arterial hypertension (PAH) [1-3]. PAH develops as a result of pulmonary vascular pathology whereas pulmonary hypertension may be secondary to severe interstitial lung disease [4]. PAH is diagnosed by right heart catheterization and pulmonary fibrosis is identified by high-resolution computed tomography (HRCT) of the chest. Increased pulmonary vascular pressures and progressive pulmonary fibrosis, if left untreated, often lead to right heart failure and eventually death. Therefore, early detection of pathological changes in the lungs is important in order to stall the progress of disease by medical therapy. The benefit of early detection of PAH has been exemplified by the improvement in haemodynamics and survival in a screening cohort compared to a detection cohort of SSc patients [5]. Since the introduction of medical treatment with angiotensin-converting enzyme inhibitors in SSc with renal involvement, pulmonary involvement is now the leading cause of death in SSc [6]. Furthermore, pulmonary fibrosis, which may occur in the absence of skin lesions, [7] may lead to an impaired gas exchange due to altered physiology in the alveolae. This impairment can be measured by studying the diffusion capacity for carbon monoxide in the lungs (DL_CO_) [8].

Cardiovascular magnetic resonance (CMR) has proven to be a highly accurate and precise tool for flow quantification and detection of small changes in blood flow [9]. Recent developments in CMR include the ability to quantify the pulmonary blood volume (PBV), as well as the variation in PBV throughout the cardiac cycle, also called the PBV variation (PBVV) normalized to the stroke volume in the pulmonary trunk (PBVV/SV). During systole, the pulmonary blood volume will increase due to the Windkessel effect. The stiffer the blood vessels, the lower the pulmonary blood volume variation. Measuring the distensibility in the pulmonary trunk alone will render information about the distensibility status of the proximal vessels, whereas the rest of the pulmonary vasculature will not be accounted for. By comparison, PBVV/SV is a measure of the global pulmonary vascular distensibility, and it is not related to change in pulmonary artery cross-sectional area [10,11]. However, little is known about how these measures are affected by pulmonary involvement such as that which occurs in SSc. It has been suggested that SSc can lead to a hyper-reactivity in the pulmonary vessels similar to the Raynaud’s phenomenon seen in the peripheral circulation [12]. However, a study assessing pulmonary haemodynamics during right heart catheterization and infusion of cold liquids showed no presence of cold induced vasoconstriction in SSc, and concluded that the constriction is rather caused by proliferation within the pulmonary arterioles [13]. This constriction in the pulmonary vessels could theoretically yield a smaller PBV in both absolute measures and indexed to lung volume (PBVI). Notably, data are not available to support this hypothesis. Furthermore, the PBV has previously not been studied in healthy subjects using CMR. Since an early detection of pulmonary involvement may have implication for patient care, the aim of this study was to explore the clinical usefulness of novel CMR techniques studying the pulmonary blood pool, including the PBV, and the PBV variation (PBVV) throughout the cardiac cycle using CMR, in SSc and healthy controls.

We hypothesized that, compared to healthy individuals, patients with SSc would have a decreased PBVI due to arteriolar involvement, and a lower PBVV/SV as a result of decreased pulmonary vascular distensibility. Therefore, the aim of this study was to prospectively explore the PBV, PBVI, and PBVV in healthy controls and patients with newly diagnosed SSc using CMR.

## Methods

### Study design

Twenty-seven consecutive newly diagnosed SSc patients, presenting to the Department of Rheumatology, Lund University Hospital, Lund and 10 healthy volunteers underwent CMR using a 1.5-T scanner (Intera, Philips, Best, the Netherlands) and a five-element cardiac coil. Out of 27 patients, 18 (67%) were diagnosed with limited cutaneous SSc, and three (11%) with diffuse cutaneous SSc. Six patients (22%) fulfilled the criteria for early SSc suggested by LeRoy [14]. Disease duration was calculated from first non-Raynaud's manifestation. The healthy volunteers were recruited by local advertisement. All volunteers had a normal electrocardiogram and did not have any previous cardiac history or cardiopulmonary medication. The study was approved by the Lund University Human Subjects Research Ethics Committee, and all subjects provided written informed consent. All image analysis was performed using the Segment software which is freely available for research use (Segment v1.8, Medviso, Lund, Sweden, http://segment.heiberg.se) [15].

### Pulmonary transit time

**Figure 1:**
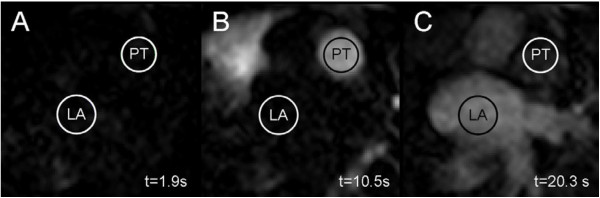
**Pulmonary transit time (PTT).** Time-resolved atrial short-axis SSFP CMR images (TR/TE 2.5/1.2 ms; flip angle 50°) bisecting the pulmonary trunk (PT) and the left atrium (LA) acquired during the first pass of an intravenous bolus of CMR contrast agent (Gd-DOTA). Panels **A**-**C** show the passage for 2 mL contrast bolus through the PT to the LA. Contrast intensity was measured in a region of interest in the PT and the LA, respectively. Time (t) is shown in seconds (s) in the lower corner on right hand side of the respective panel. **A**: time of contrast bolus injection, **B**: contrast bolus passing through PT, and **C**: arrival in LA. Note the change in signal intensity over time in the respective regions of interest.

**Figure 2:**
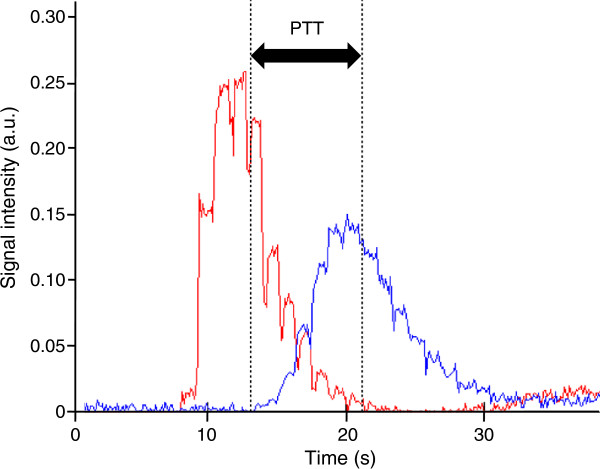
**Measurement of the pulmonary transit time (PTT).** The plot shows signal intensity (in arbitrary units, a.u.) over time for regions of interest in the pulmonary trunk (red) and the left atrium (blue), respectively. The black dotted vertical lines indicate the center of gravity for the respective curve. The double-headed arrow indicates the PTT.

The pulmonary transit time (PTT) was defined as the time for a 2 ml intravenously administered contrast bolus (gadoterate meglumine, 279.3 mg/ml, Gd-DOTA, Dotarem, Gothia Medical, Billdal, Sweden) to pass from the pulmonary trunk to the left atrium (Figures 1 and 2). The contrast, followed by 20 ml of saline, was injected at 4 ml/s using a power injector. Images were acquired in an atrial short-axis plane bisecting the left atrium and the pulmonary trunk using a saturation recovery steady-state free precession imaging sequence. Typical CMR parameters were: slice thickness 20 mm, pixel size 1.3 × 1.3 mm, temporal resolution 130 ms, TR/TE: 2.5/1.2 ms, flip angle 50°. The PTT was defined as the time between the time points of the weighted mean for each time-intensity curve, respectively, as previously validated [11].

### Pulmonary blood volume

The pulmonary blood volume (PBV) was measured as the product of the PTT and cardiac output, as previously described and validated [11]. Cardiac output was assessed by CMR flow measurement of a cross section of the pulmonary trunk or aorta using a non-segmented phase contrast velocity-encoded gradient echo sequence with retrospective ECG triggering and established analysis methods [10]. Typical CMR parameters were: slice thickness 6 mm, frames per cardiac cycle: 35, TR/TE: 8.7/5.3 ms, flip angle 15°, pixel size 1.2 × 1.2 mm, velocity encoding gradient 200 cm/s.

### Pulmonary blood volume variation

**Figure 3:**
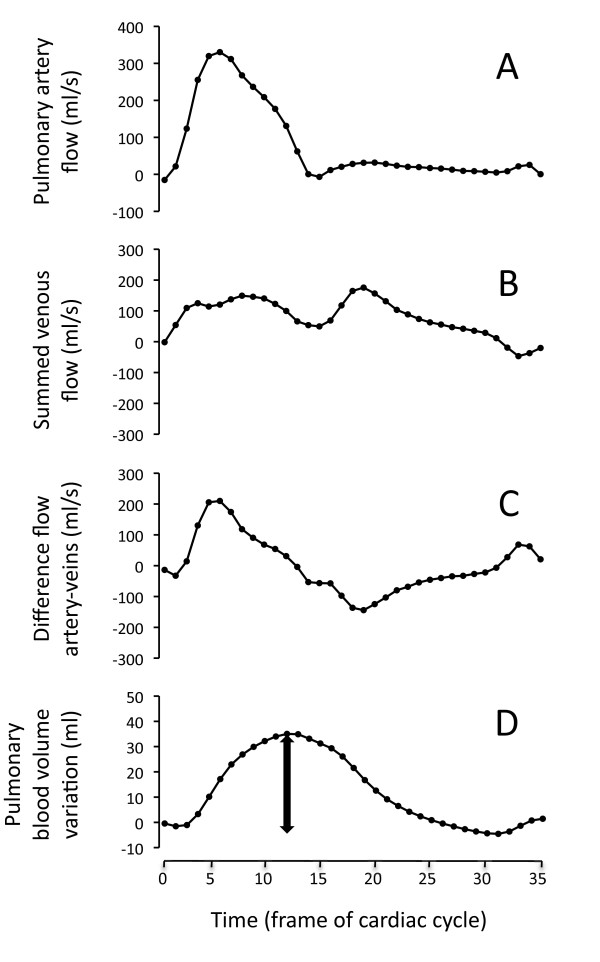
**Pulmonary blood volume variation (PBVV).** This figure shows the calculation of the pulmonary blood volume variation (PBVV) in a representative patient. Panel **A** shows the flow in the pulmonary trunk. Panel **B** shows the summed flow in all pulmonary veins. Panel **C** shows the difference in arterial and total venous blood flow. Panel **D** shows the integral of panel **C**, yielding the cumulative volume change over time in the pulmonary circulation. The PBVV was defined as the maximum change over the cardiac cycle, indicated by the double-headed arrow in Panel **D**.

Flow measurement of the pulmonary trunk and all the pulmonary veins, respectively, were acquired as previously described (Figure 3, panel A and B) [10]. The difference in arterial and venous blood flow over time (Figure 3, panel C) was integrated, yielding the cumulative blood volume in the pulmonary circulation (Figure 3, panel D). The PBV variation (PBVV) was defined as the difference between the maximum and the minimum of the cumulative volume variation over the cardiac cycle. Arterial and venous pulmonary blood flow was obtained using the same sequence as for cardiac output.

### Pulmonary blood volume indexed to lung volume

**Figure 4:**
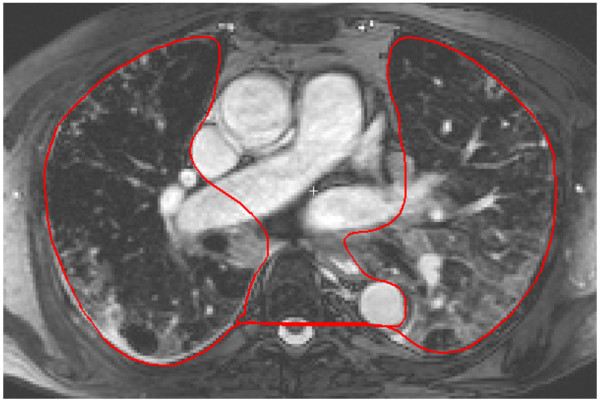
**Assessment of the pulmonary volume.** Transversal SSFP CMR was undertaken covering the entire lung field with the patient in functional end-expiratory state. The pulmonary volume was measured by planimetry in each slice, typically 50–60 slices. The figure illustrates a single representative transverse slice through the thorax. The red region of interest illustrates the region included in the pulmonary volume in this slice. The delineated area includes the functional residual capacity, the pulmonary parenchyma, and the pulmonary vessels within the lung parenchyma, but excluding the greater proximal pulmonary vessels and the aorta.

The pulmonary blood volume indexed to lung volume (PBVI) was defined as PBV/pulmonary volume. The pulmonary volume was measured by manual planimetry in a transverse SSFP CMR image stack covering the lungs during end-expiratory breathhold (Figure 4). Typical imaging parameters were: number of slices 50–60, slice thickness 5 mm, TR/TE: 3.2/1.6 ms, flip angle 80°, pixel spacing 1.4 × 1.4 mm, temporal resolution (duration of acquisition per image): 400 ms.

### Left and right ventricular functional parameters

Left and right ventricular end diastolic volume, end systolic volume, stroke volume, and ejection fraction were measured by CMR and manual planimetry using established techniques [16]. In patients, right ventricular endocardical delineation was performed in the axial plane, in the healthy controls right ventricular delineation was performed in the short-axis plane. Images were acquired using a cine SSFP sequence. Typical CMR parameters included: TR/TE: 2.9/1.5 ms, flip angle: 60°, frames per cardiac cycle: 30, reconstructed pixel size: 1.4 × 1.4 mm with an acquisition resolution of 2.1 × 2.3 mm, slice thickness: 8 mm, no slice gap, temporal resolution: 40 ms. Body surface area was calculated using the Mosteller formula [17].

### HRCT, echocardiography, and lung function

HRCT was performed using a Philips Brilliance 16-slice or 40-slice CT system (Philips, Best, The Netherlands). Data was visually assessed with regards to the presence of fibrosis-related pathology. Fibrosis was defined by the presence of traction bronchiectasis within areas of ground-glass opacity, and reticulations [18]. Patients were compared with regards to presence or absence of ground-glass opacity and/or reticulations and/or traction bronchiectasis by HRCT.

Echocardiography included Doppler measurement of the maximum velocity gradient across the tricuspid valve, and assessment of inferior vena cava size and variation in diameter during respiration. Central venous pressure was estimated in increments of 5, 10, or 15 mmHg by an experienced observer based on caval physiology. Systolic pulmonary artery pressure was calculated using the tricuspid velocity gradient and caval physiology [19] using a Philips Sonos 7500 system (Soma Technology Inc, Bloomfield, CT, USA). The upper limit by echocardiography for PAH was 30 mmHg. Patients with suspected PAH also underwent invasive measurement. An invasive mean pulmonary arterial pressure of ≥25 mmHg, pulmonary vascular resistance ≥ 3.0 Woods units, and capillary wedge pressure < 15 mmHg was defined as PAH.

Pulmonary function testing included assessment of the vital capacity (VC) and the diffusion capacity for carbon monoxide (DL_CO_) by the single-breath test.

### Statistics

Comparisons were performed using GraphPad 6.0 for Windows (GraphPad Software, Inc, La Jolla, CA, USA). Following visual inspection of the data, comparisons between groups was tested using the parametric unpaired t-test. Differences between groups were also tested using non-parametric tests and this yielded results with identical levels of significance in all comparisons. Linear regression analysis was performed with Pearson’s correlation coefficient (r). p < 0.05 was deemed statistically significant. Data are presented as mean ± SD.

## Results

Subject characteristics are described in Table 1.

**Table 1:** Subject characteristics

**Characteristics**	**SSc**	**Controls**
Total (n)	27	10
Male:female ratio	3:6	3:7
Age (y)	57 ± 13	54 ± 9
Body surface area (BSA, m^2^)	1.81 ± 0.16	1.78 ± 0.21
Heart rate (bpm)	76 ± 11	64 ± 7 †
Cardiac index (L/min/m^2^)	3.3 ± 0.8	3.5 ± 0.6
LV stroke volume index (mL/m^2^)	44 ± 11	54 ± 7
LV end diastolic volume index (mL/m^2^)	72 ± 16	89 ± 10
LV end systolic volume index (mL/m^2^)	29 ± 9	35 ± 4
LV ejection fraction (%)	61 ± 8	60 ± 3
RV stroke volume index (mL/m^2^)	42 ± 8	53 ± 7 ‡
RV end diastolic volume index (mL/m^2^)	79 ± 23	83 ±11
RV end systolic volume index (mL/m^2^)	37 ± 22	30 ± 7
RV ejection fraction (%)	55 ± 13	64 ± 6
Duration of disease (y)	6 ± 8	
Limited cutaneous SSc (n (%))	18 (67)	
Diffuse cutaneous SSc (n (%))	3 (11)	
Early SSc (n (%))	6 (22)	
PAH (n (%))	1 (4)	

LV denotes left ventricle, RV denotes right ventricle. Bpm denotes beats per minute. Disease duration was calculated from first non-Raynaud's manifestation. Early SSc denotes patients fulfilling the LeRoy criteria. PAH denotes pulmonary arterial hypertension verified by right heart catheterization (defined as mPAP >25 mmHg). Data are presented as mean ± SD. † denotes p < 0.01, and ‡ denotes p < 0.001 compared to patients with systemic sclerosis (SSc).

### The pulmonary blood volume, pulmonary blood volume index, and pulmonary blood volume variation by CMR

**Figure 5:**
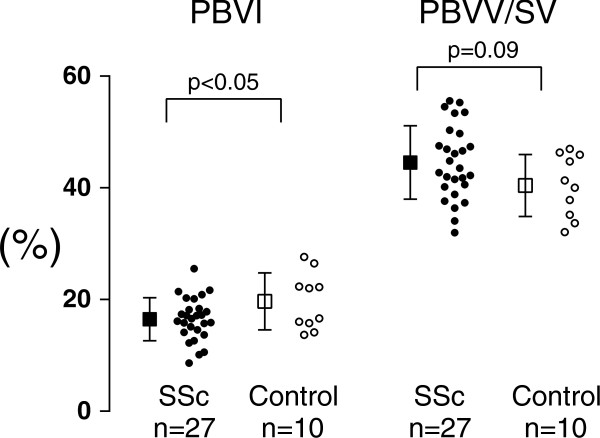
**PBVI and PBVV/stroke volume.** Results for the pulmonary blood volume indexed to the lung volume (PBVI) and the pulmonary blood volume variation divided by the stroke volume (PBVV/SV), respectively, in percent. Open boxes and circles represent healthy controls (n = 10), and black boxes and circles represent SSc patients (n = 27). Circles represent individual data and boxes and error bars show mean ± SD. Note that there was no difference between healthy controls and SSc with regards to PBVV/stroke volume.

**Figure 6:**
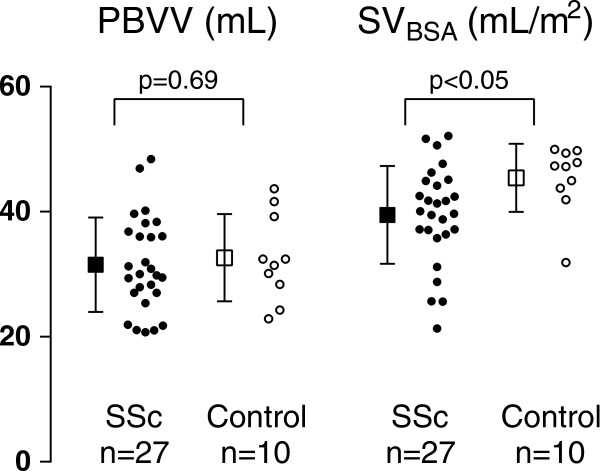
**PBVV and stroke volume.** Results for the pulmonary blood volume variation (PBVV) and stroke volume (SV), respectively, in mL and mL/m^2^, respectively. Open circles and boxes represent healthy controls (n = 10), and black circles and boxes represent SSc patients (n = 27). Circles represent individual data and boxes and error bars represent mean ± SD.

The pulmonary blood volume indexed to lung volume (PBVI) in 27 patients compared to 10 healthy controls was 16 ± 4 vs 20 ± 5%, p < 0.05 (Figure 5). There were no difference in PBV (466 ± 87 vs 471 ± 122 mL, p = 0.91) or PBVV (32 ± 8 vs 33 ± 7 mL, p = 0.69, Figure 6). The LV stroke volume indexed to BSA in 27 SSc patients was lower compared to 10 healthy individuals (44 ± 11 vs 54 ± 7 mL/m^2^, p < 0.05, respectively) (Figure 6). There was no difference in PBVV/stroke volume in patients compared to controls (45 ± 10 vs 40 ± 6, p = 0.09, (Figure 5)). There was no correlation between PBVI and LV or RV function parameters. There were no correlation between PBVI and PBVV (patients: r = 0.05, p = 0.81; controls r = -0.02 p = 0.96; for all: r = 0.05, p = 0.77).

### Echocardigraphy

Twenty-three patients (85%) had a suitable trace. There was no correlation between the estimated systolic pulmonary artery pressure assessed by Doppler echocardiography, and the PBVI (p = 0.08, n = 23). The estimated systolic pulmonary arterial pressure ranged between 26–94 mmHg.

### HRCT

Fifteen out of 24 patients (63%) were classified as having pulmonary fibrosis-related pathology by HRCT. Patients with pulmonary fibrosis by HRCT did not differ from those without pulmonary pathology with regards to PBVI (17 ± 3 vs 17 ± 5%, p = 0.74) or PBVV/stroke volume (45 ± 7 vs 44 ± 7%, p = 0.86).

### Pulmonary function test

The DL_CO_ was assessed in 23 out of 27 patients (85%) and the VC was assessed in 25 out of 27 patients (93%). There were no correlation between the PBVI and DL_CO_ or VC (p = 0.09 and p = 0.45, respectively).

#### Interobserver variability

Two observers independently evaluated 10 randomly selected patient data sets. The interobserver variability for pulmonary volume, PBV, PBVI, and PBVV was 1 ± 1%, 4 ± 12%, 2 ± 12% and 1 ± 6%, respectively.

## Discussion

The major finding in this study is that patients with newly diagnosed SSc, as a group, have a reduced amount of blood in the pulmonary vasculature normalized to lung size (PBVI) but an unaffected pulmonary vascular distensibility (PBVV/stroke volume), compared to healthy controls. There was no difference in absolute PBV between the groups. Furthermore, there was no relationship between PBVI and systolic pulmonary arterial pressure estimated by Doppler echocardiography. It remains unclear what the mechanisms are, however, our findings are compatible with previously suggested pathophysiological changes in the pulmonary vasculature [12]. Doppler-estimated pulmonary artery pressure, DL_CO_, and presence of pulmonary fibrosis-related pathology measured either on HRCT or as reduced VC did not influence PBVI. Early pathophysiological changes in the pulmonary circulation may be the reason why patients with newly diagnosed SSc differ in PBVI compared to healthy individuals. Such changes may be important to recognize as a tool to select patients eligible for right heart catheterization since an early diagnosis of PAH is paramount to improve survival in SSc.

### PBV

The current study is, to our knowledge, the first to measure the PBV by CMR in healthy controls and patients. The PBV has previously been assessed only in valvular heart disease using dye-dilution based techniques during invasive cardiac catheterization, radioactive isotope injection or echocardiography [20-24]. However, catheterization is associated with complications including perforation of great vessels, and echocardiography is both user-dependent and has limited precision when assessing cardiac output [25].

### PBVV and stroke volume

There was a lower absolute stroke volume in SSc patients compared to healthy controls. This difference may be related to myocardial disease in the SSc patients. However, there was no difference in pulmonary vascular distensibility (PBVV/stroke volume) when comparing the groups. Previously, the PBVV/stroke volume has been shown to decrease in pigs that developed increased pulmonary arterial pressure following experimental acute myocardial infarction [11]. It appears that the changes in pulmonary arterial pressure in our cohort of SSc patients either were not of a magnitude sufficient to influence PBVV, or may have been the result of a different pathophysiological process compared to experimental acute myocardial infarction. PBVV has previously been measured using nitrous oxide body plethysmography or catheter-based thermodilution techniques [26,27]. The currently presented technique using CMR has the advantage that it is non-invasive, and, in contrast to plethysmography, measures the PBVV in both the arterial and the venous vessels.

### HRCT and DL_CO_

Pulmonary fibrosis-related pathology seen on HRCT may lead to non-perfused pulmonary volumes [18]. This, per se, could lead to a decrease in the PBVI. However, there was no difference in PBVI when comparing the presence or absence of HRCT-verified fibrosis-related pathology. This suggests that PBVI measures a pathophysiological mechanism independent of pulmonary fibrosis. In the very first descriptions of SSc patients developing PAH, a declining DL_CO_ but normal VC was a key feature. However, there was no correlation between the PBVI and DL_CO_, which suggests that the PBVI may reflect pulmonary involvement prior to changes severe enough to alter DL_CO_.

### Interpreting the PBVI

The SSc patients in the present study displayed a wide range of PBVI values. This may be because the patients are in different stages of disease. However, some patients even had a higher value than the controls. This may be related to left ventricular dysfunction, leading to backward failure and an increased amount of blood in the pulmonary circulation. There was no correlation between estimated pulmonary artery pressure and PBVI. Previously, catheter-based techniques have been used to show an increased PBV in patients with stenosis or regurgitation in the mitral valve, [22,28] presumably due to backward failure and distension of the pulmonary venous and/or pulmonary arterial blood pool. Consequently, the following pathophysiological mechanisms may apply to patients with SSc. On the one hand, pulmonary arteriolar proliferation may reduce the PBV, presumably on the arterial side, while on other hand, backward failure, if present, may increase the PBV, presumably on the venous side. Thus, SSc patients with a decreased arterial blood pool and simultaneous backward failure could hypothetically present with a PBVI within normal values or higher.

### Limitations

One limitation of the study may be the accuracy of measuring PBVV, since the PBVV is derived from up to 6 different flow measurements comprised of one measurement each from the pulmonary artery and at most 5 pulmonary veins, respectively. However, the interobserver variability between two experienced observers was low, and a previous study with identical methodology has shown good correspondence between the volumes of measured in- and outflow from the pulmonary circulation [10] and excellent agreement between flow-derived and planimetrically derived volume changes in the heart [9]. Furthermore, the comparison between the PBVI and PBVV and pulmonary arterial pressures were made using Doppler-echocardiography derived pressures. Invasive pressures were only acquired in three patients with suspected PAH, and this is a limitation. Another limitation is that there currently is no gold standard to measure the pulmonary volume including the functional end-expiratory lung volume. Thus, a future comparison between CMR and computed tomography may be of value to address this issue. Moreover, test-retest variability would be of value to perform and this has not been done.

## Conclusions

Compared to healthy controls, patients with newly diagnosed SSc have, as a group, a reduced amount of blood in the pulmonary vasculature (PBVI) but an unaffected pulmonary vascular distensibility (PBVV/stroke volume), not related to the presence of pulmonary fibrosis-related pathology on HRCT, estimated pulmonary artery pressure, DL_CO_ or reduced VC. This implies that the PBVI may reflect the morphology of the SSc lung since the majority of the patients did not show any clinical sign of pulmonary involvement. These findings are consistent with an involvement of pulmonary arterioles in SSc. Therefore, in the case of reduced PBVI, an earlier medical treatment may be indicated in order to stall the progress of further pulmonary involvement. Also, this study shows the feasibility of assessing the PBV in healthy individuals and clinical SSc patients by using CMR. Further studies are justified to assess the clinical utility of PBVI as a non-invasive diagnostic and prognostic measure of pathophysiological changes in the pulmonary circulation.

## Competing interests

The authors declare that they have no competing interests.

## Authors’ contributions

MK participated in data acquisition, performed the image analysis and data analysis, and drafted the manuscript. DMW participated patient recruitment. GB performed computed tomography image analysis. RH performed data acquisition, and coordinated patient recruitment. MU conceived the study, participated in data analysis, drafting the manuscript, and had full access to and was responsible for the integrity of the data. All authors participated in the design of the study, critically revised the manuscript for important intellectual content, and approved the final manuscript.

## References

[B1] Hachulla E Gressin V Guillevin L Carpentier P Diot E Sibilia J Kahan A Cabane J Francès C Launay D Early detection of pulmonary arterial hypertension in systemic sclerosis: a French nationwide prospective multicenter study Arthritis Rheum 2005 52 12 3792 3800 10.1002/art.21433 16320330

[B2] Wigley FM Lima JAC Mayes M McLain D Chapin JL Ward-Able C The prevalence of undiagnosed pulmonary arterial hypertension in subjects with connective tissue disease at the secondary health care level of community-based rheumatologists (the UNCOVER study) Arthritis Rheum 2005 52 7 2125 2132 10.1002/art.21131 15986394

[B3] Vonk MCBB Heijdra YF Ton E Snijder R van Dijk APJ van Laar JM Bootsma H van Hal PTW van den Hoogen FHJ van Daele PLA Systemic sclerosis and its pulmonary complications in The Netherlands: an epidemiological study Ann Rheum Dis 2009 68 6 961 965 10.1136/ard.2008.091710 18511546

[B4] Opie LH The pulmonary manifestations of generalised scleroderma (progressive systemic sclerosis) Dis. Chest 1955 28 6 665 680 10.1378/chest.28.6.665 13270668

[B5] Humbert M Yaici A de Groote P Montani D Sitbon O Launay D Gressin V Guillevin L Clerson P Simonneau G Hachulla E Screening for pulmonary arterial hypertension in patients with systemic sclerosis: clinical characteristics at diagnosis and long-term survival Arthritis Rheum 2011 63 11 3522 3530 10.1002/art.30541 21769843

[B6] Steen VD Medsger TA Changes in causes of death in systemic sclerosis, 1972–2002 Ann Rheum Dis 2007 66 7 940 944 10.1136/ard.2006.066068 17329309 PMC1955114

[B7] Rodnan GP Fennell RH Jr Progressive systemic sclerosis sine scleroderma JAMA 1962 180 665 670 10.1001/jama.1962.03050210027006 14493142

[B8] Blakemore WS Forster RE Morton JW Ogilvie CM A standardized breath holding technique for the clinical measurement of the diffusing capacity of the lung for carbon monoxide J Clin Invest 1957 36 1 Part 1 1 17 13398477 10.1172/JCI103402 PMC1087291

[B9] Carlsson M Cain P Holmqvist C Stahlberg F Lundback S Arheden H Total heart volume variation throughout the cardiac cycle in humans Am J Physiol Heart Circ Physiol 2004 287 1 H243 250 10.1152/ajpheart.01125.2003 15016625

[B10] Ugander M Jense E Arheden H Pulmonary intravascular blood volume changes through the cardiac cycle in healthy volunteers studied by cardiovascular magnetic resonance measurements of arterial and venous flow J Cardiovasc Magn Reson 2009 11 42 10.1186/1532-429X-11-42 19878570 PMC2773236

[B11] Ugander M Kanski M Engblom H Gotberg M Olivecrona GK Erlinge D Heiberg E Arheden H Pulmonary blood volume variation decreases after myocardial infarction in pigs: a quantitative and noninvasive MR imaging measure of heart failure Radiology 2010 256 2 415 423 10.1148/radiol.10090292 20656833

[B12] Sackner MA The visceral manifestations of scleroderma Arthritis Rheum 1962 5 184 194 10.1002/art.1780050207 14495869

[B13] Mukerjee D Yap LB Ong V Denton CP Howells K Black CM Coghlan JG The myth of pulmonary Raynaud's phenomenon: the contribution of pulmonary arterial vasospasm in patients with systemic sclerosis related pulmonary arterial hypertension Ann Rheum Dis 2004 63 12 1627 1631 10.1136/ard.2003.015289 15547087 PMC1754861

[B14] LeRoy EC Medsger TA Jr Criteria for the classification of early systemic sclerosis J Rheumatol 2001 28 7 1573 1576 11469464

[B15] Heiberg E Sjogren J Ugander M Carlsson M Engblom H Arheden H Design and validation of segment–freely available software for cardiovascular image analysis BMC Med Imaging 2010 10 1 10.1186/1471-2342-10-1 20064248 PMC2822815

[B16] Pennell DJ Ventricular volume and mass by CMR J Cardiovasc Magn Reson 2002 4 4 507 513 12549238 10.1081/jcmr-120016389

[B17] Mosteller RD Simplified calculation of body-surface area N Engl J Med 1987 317 17 1098 3657876 10.1056/NEJM198710223171717

[B18] Remy-Jardin M Giraud F Remy J Copin MC Gosselin B Duhamel A Importance of ground-glass attenuation in chronic diffuse infiltrative lung disease: pathologic-CT correlation Radiology 1993 189 3 693 698 8234692 10.1148/radiology.189.3.8234692

[B19] Yock PG Popp RL Noninvasive estimation of right ventricular systolic pressure by doppler ultrasound in patients with tricuspid regurgitation Circulation 1984 70 4 657 662 10.1161/01.CIR.70.4.657 6478568

[B20] Donato L Giuntini C Lewis ML Durand J Rochester DF Harvey RM Cournand A Quantitative radiocardiography. I. Theoretical considerations. Circulation 1962 26 174 182 13887190 10.1161/01.cir.26.2.174

[B21] Giuntini C Lewis ML Luis AS Harvey RM A study of the pulmonary blood volume in man by quantitative radiocardiography J Clin Invest 1963 42 1589 1605 10.1172/JCI104844 14077387 PMC289438

[B22] McGaff CJ Roveti GC Glassman E Milnor WR Pulmonary blood volume in rheumatic heart disease and its alteration by isoproterenol Circulation 1963 27 77 84 10.1161/01.CIR.27.1.77 17894029

[B23] Milnor WR Jose AD McGaff CJ Pulmonary vascular volume, resistance, and compliance in man Circulation 1960 22 130 137 10.1161/01.CIR.22.1.130 14422672

[B24] Mischi M Kalker TA Korsten EH Contrast echocardiography for pulmonary blood volume quantification IEEE Trans Ultrason Ferroelectr Freq Control 2004 51 9 1137 1147 15478975 10.1109/tuffc.2004.1334846

[B25] Ehler D Carney DK Dempsey AL Rigling R Kraft C Witt SA Kimball TR Sisk EJ Geiser EA Gresser CD Guidelines for cardiac sonographer education: recommendations of the American society of echocardiography sonographer training and education committee J Am Soc Echocardiogr 2001 14 1 77 84 10.1067/mje.2001.109922 11174441

[B26] Her C Hayes D Lees DE Elevated pulmonary artery systolic storage volume associated with redistribution of pulmonary perfusion Crit Care Med 1987 15 11 1023 1029 10.1097/00003246-198711000-00007 3677744

[B27] Karatzas NB Lee GJ Propagation of blood flow pulse in the normal human pulmonary arterial system. Analysis of the pulsatile capillary flow. Circulation research 1969 25 1 11 21 10.1161/01.RES.25.1.11 5795012

[B28] Roy SB Bhardwaj P Bhatia ML Pulmonary blood volume in mitral stenosis Br Med J 1965 2 5476 1466 1469 10.1136/bmj.2.5476.1466 5849437 PMC1847055

